# Supplementary material for: A genetic perspective on the relationship between eudaimonic –and hedonic well-being
Source: Sci Rep. 2018 Oct 2;8:14610. doi: 10.1038/s41598-018-32638-1 (PMC6168466; doi:10.1038/s41598-018-32638-1)

**A genetic perspective on the relationship between**

**eudaimonic –and hedonic**

**well-being**

B.M.L. Baselmans^1, 2^, M.Bartels^1, 2, 3*^

**Supplementary Figures**

**^1^ Departement of Biological Psychology, Vrije Universiteit, Amsterdam.**

^2^ Amsterdam Public Health Institute, Amsterdam, the Netherlands

^3^ Neuroscience Amsterdam, Amsterdam, the Netherlands

* Corresponding Author

Bart Baselmans

Department of Biological Psychology

Vrije Universiteit Amsterdam

The Netherlands

[b.m.l.baselmans@vu.nl](mailto:m.bartels@vu.nl)

**Figure 1:** Manhattan plot for the univariate GWAS results of Hedonic Well-being (UKB ID 4526)

**Figure 2:** Manhattan plot for the univariate GWAS results of Hedonic Well-being (UKB ID 20458)

**Figure 3:** Polygenic scores including 10 *P*-value thresholds for Eudaimonic and Hedonic Well-being

**Figure 4:** Cell type specific enrichment for eudaimonic –and hedonic well-being

**Supplementary Figure 1:** Manhattan Plot for GWAS results. Result is shown for Hedonic well-being (UKB ID 4526). The *x* axis shows chromosomal position, and the *y* axis shows association significance on a −log10 scale. The upper dashed line marks the threshold for genome-wide significance (*P* = 5 × 10−8), and the lower dashed line marks the threshold for nominal significance (*P* = 1 × 10−5). Each approximately independent genome-wide significant association (lead SNP) is marked by an orange **Δ**. Each lead SNP is the SNP with the lowest *P* value within the locus, as defined by our clumping algorithm


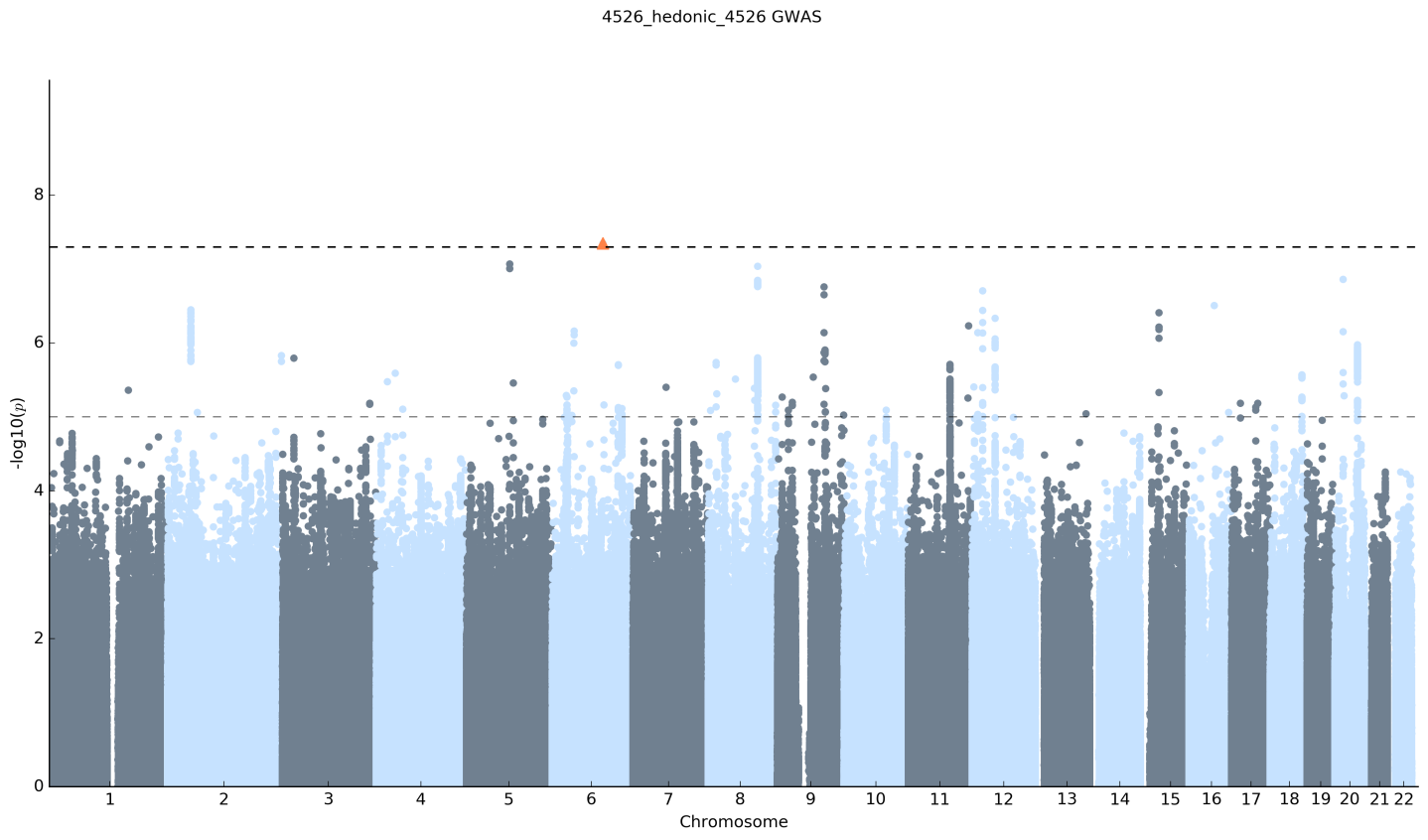


**Supplementary Figure 2:** Manhattan Plot for GWAS results. Result is shown for Hedonic well-being (UKB ID 20458). The *x* axis shows chromosomal position, and the *y* axis shows association significance on a −log10 scale. The upper dashed line marks the threshold for genome-wide significance (*P* = 5 × 10−8), and the lower dashed line marks the threshold for nominal significance (*P* = 1 × 10−5). Each approximately independent genome-wide significant association (lead SNP) is marked by an orange **Δ**. Each lead SNP is the SNP with the lowest *P* value within the locus, as defined by our clumping algorithm

**
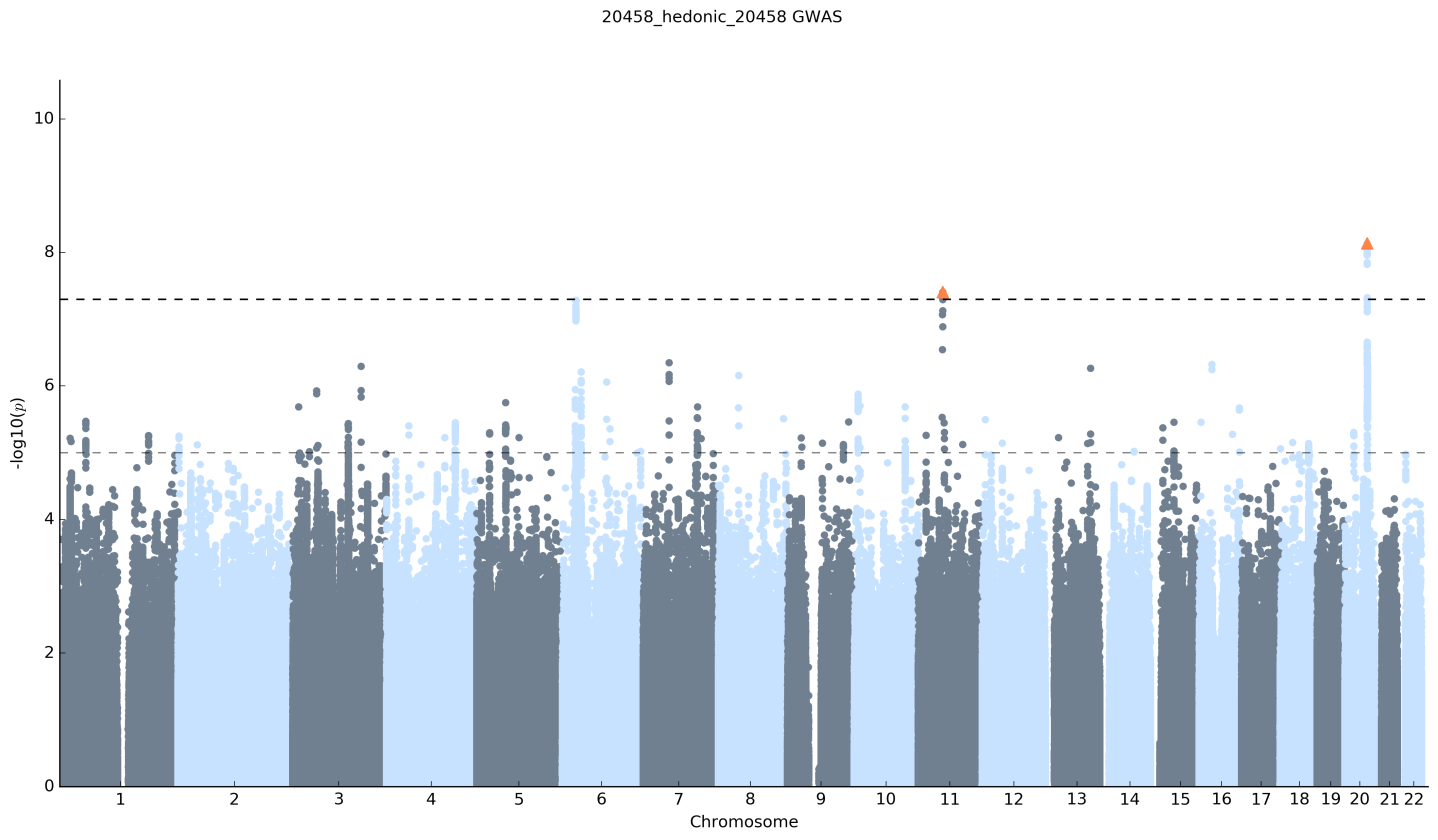
**


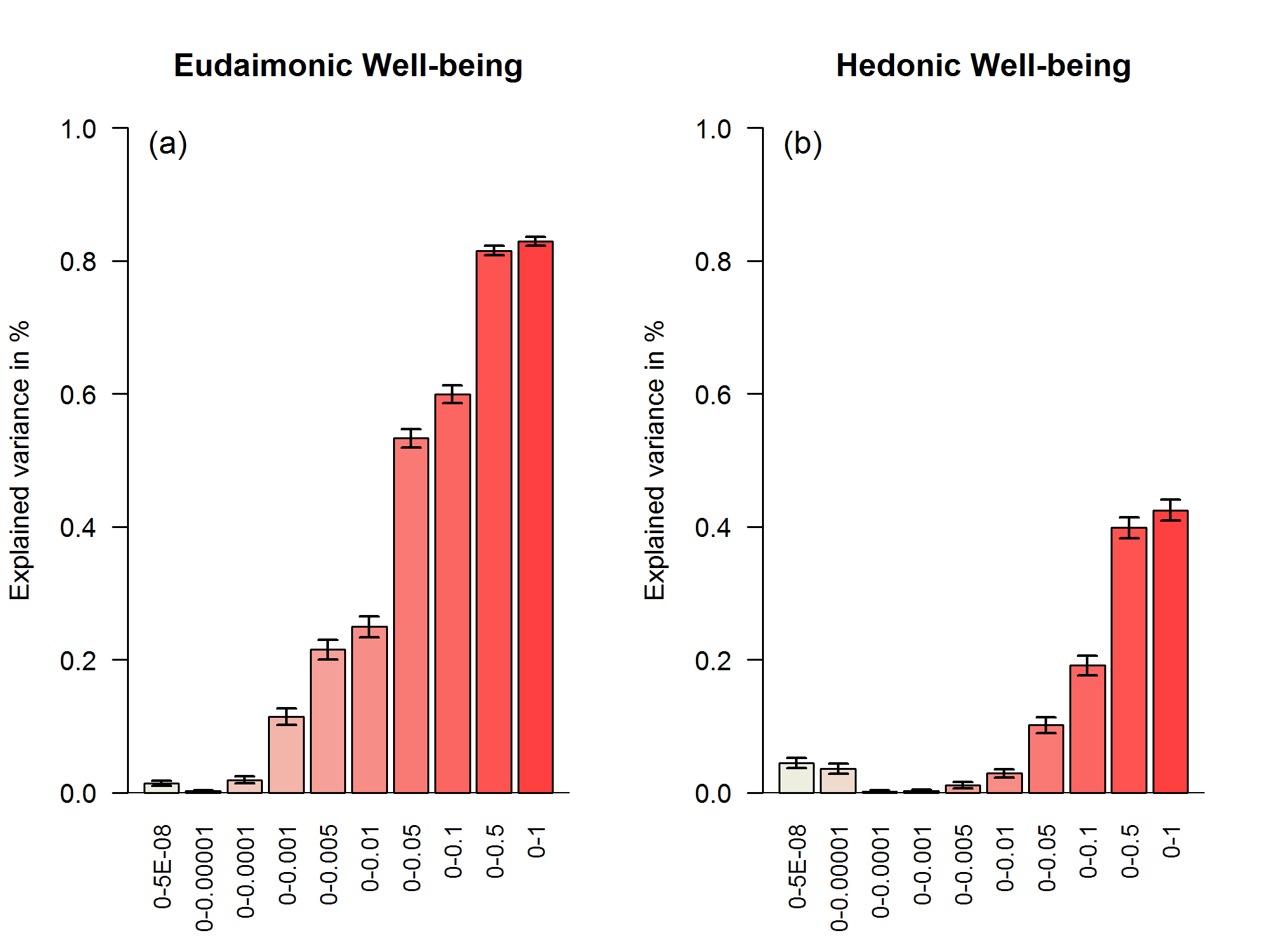
**Figure 3:** Polygenic scores using 10 different p-value thresholds for **(a)** Eudaimonic, and **(b)** Hedonic. The x axis shows the 10 different P value thresholds, the y axis shows the explained variance in percentage.

**Figure 4:** Tissue specific enrichment using 53 specific tissue types for **(a)** Eudaimonic, **(b)** Hedonic. Bar-graphs above the dashed line are significantly enriched. The x axis shows the 53 different categories whereas the y axis shows the –log^10^ P value. Bars in blue are significant enriched.


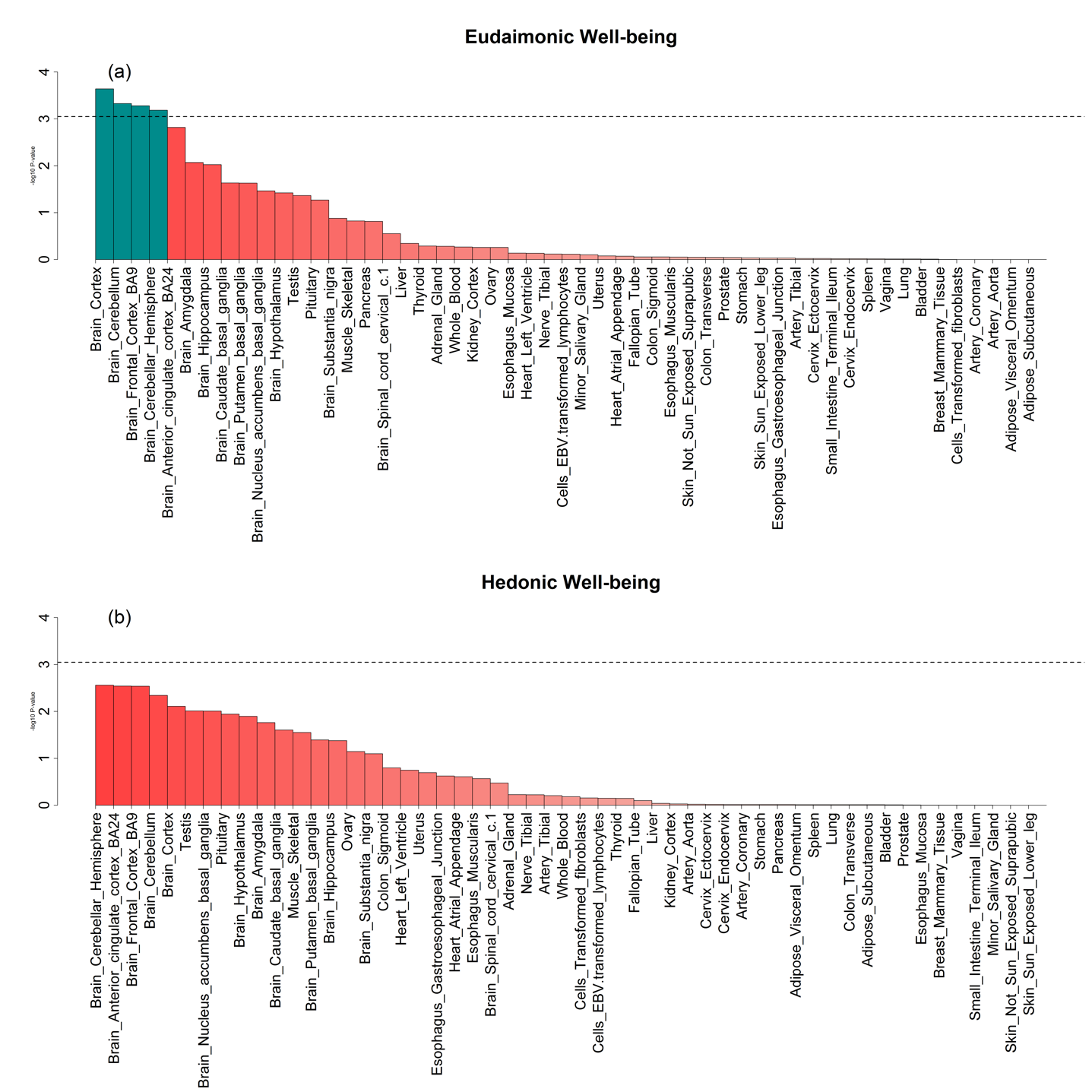

Supplement: Supplementary file 1 — Supplementary Figures [file 41598_2018_32638_MOESM1_ESM.docx]
